# Loneliness at Older Ages in Japan: Variation in Lonely Life Expectancy and the Role of Social Isolation

**DOI:** 10.1093/geroni/igab046.1779

**Published:** 2021-12-17

**Authors:** Shiro Furuya, James Raymo

**Affiliations:** 1 University of Wisconsin--Madison, Madison, Wisconsin, United States; 2 Princeton University, Princeton University, New Jersey, United States

## Abstract

Despite growing media, policy, and research attention to loneliness, it remains an understudied dimension of inequality in demography. Additionally, research on loneliness often fails, both methodologically and conceptually, to distinguish loneliness from social isolation. This is an important limitation given the positive correlation between measures of these two distinct concepts, a relationship that may be particularly relevant in collectivistic societies. This study focuses on Japan, describing the synthetic cohort duration of exposure to loneliness at older ages, with and without adjusting for the correlation between loneliness and social isolation. Combining life tables from the Human Mortality Database with individual data from the National Survey of Japanese Elderly, we calculated isolation-adjusted lonely life expectancies. We also evaluated regional and educational differences in isolation-adjusted lonely life expectancies. Results showed significant differences in lonely life expectancy before and after adjusting for social isolation; however, the attention to social isolation did little to alter our general understandings of trends and differentials in lonely life expectancy. In contrast to public perceptions of growing loneliness, we find that lonely life expectancy is short among older Japanese and has not increased over time. Additionally, we found no clear regional nor educational differences in isolation-adjusted lonely life expectancy.

